# Pediatric Selective IgM Immunodeficiency

**DOI:** 10.1155/2008/624850

**Published:** 2008-11-24

**Authors:** Marc F. Goldstein, Alex L. Goldstein, Eliot H. Dunsky, Donald J. Dvorin, George A. Belecanech, Kfir Shamir

**Affiliations:** ^1^The Asthma Center, Allergic Disease Associates, PC, 205 N. Broad street, Suite 300, Philadelphia, PA 19107, USA; ^2^Wharton Undergraduate Division ('06), University of Pennsylvania, Philadelphia, PA 19104, USA; ^3^Department of Medicine, Hahnemann University Hospital, Broad and Vine Streets, Philadelphia, PA 19102, USA

## Abstract

*Objective*. Limited information exists on features of pediatric Selective IgM immunodeficiency (SIgMID). Previously published pediatric cases and 2 new cases are reviewed. *Methods*. English literature from PubMed and references from relevant articles were reviewed. Previously reported cases and 2 new cases from an allergy/immunology practice were analyzed. *Results*. Forty-nine reported cases of SIgMID presented with respiratory infections (77.6%), gastrointestinal disease (16.3%), skin disease (12.2%), and meningitis (8.2%). Mean serum IgM level was 16.5 ± 13.8 mg/dL. Two patients were identified with SIgMID among 6300 active pediatric patients (0.03%) presenting with asthma, vasomotor rhinitis, and recurrent respiratory infections. In the 51 cases reported, none developed lymphoproliferative disease nor evolved into panhypogammaglobulinemia; four fatalities were reported. *Conclusions*. The prevalence of SIgMID in our pediatric population was 0.03%. In general, respiratory infections are the common comorbid conditions. Death and autoimmune disease are uncommon complications of pediatric SIgMID.

## 1. INTRODUCTION

Selective IgM immunodeficiency (SIgMID) is a dysgammaglobulinemia characterized by an isolated low level of serum IgM, usually <20 mg/dL in infants and children or <2 standard deviations or 10% below age adjusted means [1–3]. Usually, serum IgM levels are <10–20 mg/dL [3]. The level of other immunoglobulin isotypes is typically normal, although IgE may be increased. It is said to be a rare primary immunodeficiency, with the prevalence of completely deficient IgM patients reported as approximately 0.03% in a community-based study. [1] However, the prevalence of those with deficient but detectable levels of IgM is closer to 0.1–3.0% in hospitalized patients [1, 4, 5], 1.6% in an unselected community health screening [1, 6], 0.07% in an allergy and immunology clinic [7], and 0.26% in an adult allergy and immunology clinic [8]. There is a slightly higher penetration of SIgMID in males (1.97%) versus females (1.42%) [1]. The prevalence in the pediatric population (<18 years of age) has not been reported.

A variety of bacterial and viral infections have been linked with SIgMID in the pediatric and adult populations (Table 1) [8]. In children, infectious agents have included *Pneumocystis carinii* [9], *Giardia* [10], *Staphylococcus* [10, 11], *Salmonella* [12], *Listeria monocytogenes* [13], meningococcus [6, 14, 15], *Pseudomonas* [10, 16], molluscum contagiosum [17], cytomegalovirus [18], and varicella [17]. These organisms account for recurrent infectious dermatitis, diarrhea, meningitis, upper and lower respiratory infections, sepsis, and in some cases, death. Secondary IgMID presumptively from another cause has been associated with an array of noninfectious diseases in children and adults, in particular, autoimmune diseases and malignancies [8]. In children, celiac disease and autoimmune hemolytic anemia (Table 1) have been reported but other autoimmune diseases and malignancies have not. We present a review of 49 previously reported pediatric patients with SIgMID and 2 new cases from our practice. The relative frequencies of various clinical, immunological and demographic features, associations, and complications are established in this series. These findings are also compared and contrasted to adult cases previously reported [8].

## 2. MATERIALS AND METHODS

We undertook a retrospective (2002–2005) medical record review of 20000 charted patients seen in our practice over a 3-year period. Of these, 6300 patients were children (<18 years of age). Charts were selected with a diagnosis of SIgMID, selective IgA immunodeficiency (SIgAID), common variable immunodeficiency (CVID), Bruton agammaglobulinemia, and transient hypogammaglobulinemia of infancy and reviewed for immunoglobulin levels (IgG, IgM, IgA, IgE, and IgG subclasses), isohemagglutinin levels, autoantibody serologies, presenting clinical symptoms, concurrent conditions, and clinical course. Patients diagnosed with SIgMID were screened serologically for celiac disease, autoimmune thyroid disease, and autoimmune collagen vascular disease. A literature search was conducted of reported cases of SIgMID in the English literature through PubMed from 1966, and from the bibliographies of related articles. Identified in the literature were 361 previously reported SIgMID patients, comprised of 155 adult, 49 pediatric, and 157 age unspecified patients. The analyses of the 155 adult, 157 age-unspecified cases, and 36 new adult cases of SIgMID have been previously reported [8]. Comparative analysis was made of clinical, laboratory, and demographic data of pediatric cases to reported adult cases of SIgMID.

## 3. STATISTICAL ANALYSIS

The group mean and 1 SD were calculated for group serum IgM, IgA, IgG, IgE levels and age of presentation. Descriptive statistics were used to denote frequencies of occurrence of comorbid conditions. Statistical analyses were done with Microsoft Excel (Microsoft Corporation, Redmond, WA).

## 4. RESULTS

### 4.1. Previously reported pediatric cases

Forty-nine previously described pediatric cases of SIgMID were identified ranging in age from 1 month to 17 years (Table 1). The mean age at the time of diagnosis was 6.0 ± 4.7 years. Of these cases, where reported, presenting respiratory infections occurred in 78.4% of cases (Table 2). Frequencies of specific presenting respiratory infections were otitis media (39.2%), pneumonia/lower respiratory infections (19.6%), bronchitis (9.8%), and upper respiratory infections (9.8%). Frequency of nonrespiratory presenting illnesses included gastrointestinal illness (13.7%), skin disorders (9.8%) with atopic dermatitis the most common (6.1%), failure to thrive (7.8%), asthma (7.8%), meningitis (7.8%), and vasomotor rhinitis (3.9%). Five patients had celiac disease and serum IgM levels normalized in all cases on gluten free diets. There were no patients with malignancies reported. There were 2 asymptomatic cases reported. The mean serum IgM level was 16.5 ± 13.8 mg/dL. Serum IgA and IgG levels were normal in all cases. Mean serum IgE level was elevated at 1814 ± 3509 IU/mL. Four patients developed respiratory allergy while 3 patients developed asthma. Two patients lacked protective IgG responses to polysaccharide and protein antigen/vaccine challenge and clinically responded to intravenous immunoglobulin (IVIg) treatment. Where tested, most patients had low-to-normal titers of IgM isohemagglutinins. Infections from pneumococcus, *Staphylococcus*, *Pseudomonas*, *Salmonella*, *Pneumocystis carinii*, *Giardia*, cytomegalovirus, *Listeria monocytogenes*, meningococcus, molluscum contagiosum, and varicella were reported.

### 4.2. Pediatric cohort

*Sex and Race*: there were 2 Caucasian males and 0 females identified in our pediatric population of 6300 patients.

*Age at time of diagnosis*: age of diagnosis of SIgMID was 10 (patient 1) and 12 years of age (patient 2). Onset of SIgMID could not be determined or estimated accurately, although patient 1 had normal IgM, IgG, IgA levels 6 years prior to diagnosis. *Observation Period*: patient 1 had been followed for 8 years and patient 2 for 5 years post diagnosis. *Immunoglobulin Levels*: two patients in our pediatric patient population of 6300 (incidence 0.03%) were identified with reduced serum IgM levels less than 2 SD of age-adjusted means. Asymptomatic patients may have been missed since only symptomatic patients with recurrent infections or unusual infection were screened for immunodeficiency. Neither patient had undetectable IgM levels. The serum IgM levels were 21 mg/dL (patient 1) and 30 mg/dL (patient 2) with normal serum IgA, serum IgG levels, and IgG subclasses. The serum IgE level was 15 IU/ml in patient 1 [normal levels (<200 IU/ml)]. Immunoglobulin levels were repeated 6 months and 2, 4, and 7 (patient 1) years after diagnosis and remained essentially the same with persistence of serum IgM <30 mg/dL in both patients through time. Within the same database of patients there were no pediatric patients with CVID, Bruton agammaglobulinemia, transient hypogammaglobulinemia of infancy, or SIgAID. *Serologic Evaluation*: because of previous reports of autoimmune disease, thyroiditis, and celiac disease in patients with SIgMID, the 2 patients were screened for anemia, ANA, anti-endomysial antibody, anti-gliadin antibody, anti-transglutaminase antibody, and thyroid autoantibodies. No autoantibodies were identified in either patient. Neither patient was anemic. *Functional Antibodies*: both patients were tested for ABO blood group and isohemagglutinin level. Detectable low titer isohemagglutinins were noted (patient 1 anti-A 1:4; patient 2 anti-B 1:8). IgG antibody responses to tetanus, *H. influenzae*, and pneumococcus vaccination were normal.

**Clinical manifestations**

*Infections*: our 2 patients presented with a history of respiratory infections: patient 1 with recurrent otitis media and patient 2 with pneumonia. Neither patient had a severe life-threatening infection. Neither patient had an unusual bacterial, viral, fungal, or parasitic infection, meningitis, bacteremia, or abscess prior to or during the followup period. *Gastrointestinal Abnormalities*: chronic gastrointestinal illnesses were not noted. *Autoimmune Disease*: no autoimmune disorders were noted at time of presentation or during the followup period. *Other Respiratory Conditions including Allergy*: upper respiratory allergies were ruled out by skin testing. Other noninfectious respiratory conditions seen included asthma and vasomotor rhinitis in both patients. Neither patient had nasal polyps. *Miscellaneous Abnormalities*: neither idiopathic angioedema nor idiopathic anaphylaxis was seen. During the followup period of observation, neither patients transformed into CVID. *Mortality*: neither patient died of serious infections, malignancies or complications of autoimmune disease. *Family History of Patients*: there were no family histories of infantile deaths, increased susceptibility to infections, or primary immunodeficiencies among immediate relatives. Screenings for immunodeficiencies in parents and siblings were recommended but compliance was inconsistent. Where tested, no parent or sibling was identified with an immunoglobulin deficiency. *Therapy*: neither patient was put on IVIg therapy. In general, infections were successfully treated with conventional courses of antibiotics.

## 5. DISCUSSION

Immunoglobulin *M* is a pentamer found in the intravascular compartment and on the surface of B lymphocytes. It is the antibody isotype produced initially in the immune response, and the first immunoglobulin class to be synthesized by a fetus or newborn. IgM antibodies do not cross the placenta, and for these reasons the demonstration of IgM specific antibody is useful in the assessment of neonatal infection. Dysregulation of IgM leading to elevated or reduced serum levels has been reported in a number of conditions [8]. In children, isolated elevated levels of IgM have been associated with acute or recurrent infections (Hyper-IgM immunodeficiency, Epstein Barr virus infection) [32, 33]. Fluctuating levels of serum IgM associated with secondary conditions in some adult cases may have prognostic significance, as in adult systemic lupus erythematosus (SLE) [34]. Decreased levels of IgM have been associated with episodes of recurrent infection, thymic hypoplasia, celiac disease, autoimmune disease, and certain adult malignancies and several primary immunodeficiencies (Wiskott-Aldrich Syndrome, ataxia-telangiectasia, CVID, Bruton agammaglobulinemia, SIgMID, combined IgG and IgM immunodeficiency, and transient hypogammaglobulinemia of infancy) and congenital disorders (Bloom syndrome and Russell-Silver syndrome). Isolated reduced serum IgM levels secondary to these other illnesses should be excluded before making a diagnosis of primary SIgMID. Primary selective IgM immunodeficiency is less common than secondary IgM immunodeficiency.

SIgMID has been characterized as a rare primary immunodeficiency differentiated by a low serum IgM level, less than 2 SD or <10% of age adjusted normal controls or absolute levels of 10–20 mg/dL in infants and children [3]. The earliest recognized cases were described in children in 1966 [9, 19]. More pediatric cases and the first adult cases were described in the late 1960s and were classified as dysgammaglobulinemia V [2, 6, 12, 21]. Over the subsequent 40 years, small series and isolated case reports have appeared. Recently, a retrospective review with a large cohort of adult SIgMID was reported further characterizing this illness in patients ≥18 years of age [8].

Heterogeneous lymphocyte abnormalities have been reported in patients (mainly adults) with SIgMID. Several in vitro and in vivo immunologic, phenotypic and functional profiles have been described, some conflicting, and are summarized in Table 3.

Heterogeneous gene impairments regulating terminal B cell differentiation have been reported in SIgAID [35] and CVID. Recently, mutations in the inducible T cell costimulator gene, the transmembrane activator and calcium modulator, and cyclophilin ligand interactor have been described in CVID and SIgAID [36]. Similar in depth in vitro studies of lymphocyte function and molecular genetic studies have not been applied to pediatric and adult patients with SIgMID [8]. Functional (except isohemagglutinin titers), phenotypic or molecular genetic studies were not performed in our cohort. The latter was not the subject of this analysis.

SIgMID is regarded as an uncommon disorder and often neglected in the discussion of primary immunodeficiencies [36, 37]. A discussion of SIgMID is notably absent in recently published practice parameters on primary immunodeficiencies from expert panels [37]. The prevalence of SIgMID has been reported to be less than SIgAID in an unselected group of 3,213 individuals ages 4–87 [1]. In the atopic population, the prevalence of both SIgAID (4.5%) and SIgMID (ranging from 1.56–22%) may be higher than in the general population [38]. The prevalence of SIgMID in an adult symptomatic population of patients going to an allergy and immunology practice (1:385) [8] may be much higher than previously thought (1:15,000) [7]. By comparison, the prevalence in our pediatric symptomatic patients going to an allergy and immunology practice was 0.03%—one-tenth of the adult prevalence. Asymptomatic patients with SIgMID have been reported (up to 19% in some series) [14, 21, 39], but only 2 cases were observed in children [14, 21]. Asymptomatic pediatric patients with SIgMID were not screened for or identified in our retrospective practice database analysis. These cases are typically found during the investigation of other diseases (autoimmune disease or cancer) or in family members of patients with immunodeficiency or by chance. Screening for asymptomatic cases would be cost prohibitive. In our review, 1 pediatric patient with a complete deficiency of IgM was identified, reflecting the rarity of such an occurrence [16]. In Cassidy’s population of 3213 unselected Caucasian individuals, only 1 patient had undetectable serum IgM levels (0.03% incidence) [1].

Common with other primary immunodeficiencies, recurrent sinopulmonary infections were present in 74.5% of pediatric SIgMID cases (Table 4). Although meningitis, sepsis, atypical infections and fatalities secondary to infections have previously been reported in pediatric SIgMID, these complications were not seen in our 2 cases. In contrast to CVID and SIgAID where antibiotics may be used for extended periods along with prophylactic antibiotics, our 2 pediatric patients with recurrent respiratory infections responded to conventional courses of antibiotics and were not treated with prophylactic antibiotics. Also different from CVID and SIgAID, our patients, and the majority of previously reported pediatric cases, did not receive IVIg. Two pediatric SIgMID cases with functional IgG antibody deficiency received IVIg [31]. Given these observations, adult patients with SIgMID and most pediatric cases of SIgMID experience infections with conventional organisms. The absence of virulent infections in most cases may be due to effective antibiotic therapy and/or the response of other immune systems to microorganisms that may compensate for the low level of IgM. In addition, earlier reported fatalities from infections (all prior to 1972) may reflect less effective antibiotic coverage and/or hospital care than that currently available. Nevertheless, like other primary immunodeficiencies. the morbidity of frequent infection in SIgMID is high. Appropriate immunization (influenza, *H. influenzae*, pneumococcus, pertussis), attention to concomitant treatment directed at allergic inflammation, and good hygiene are important preventative measures. Aggressive antimicrobial therapy is recommended to prevent and manage infectious complications. IVIg may be instituted in cases of recurrent, debilitating or life threatening infection, and/or in patients with concomitant functional IgG deficiencies.

Autoimmune phenomena are seen in association with several immunoglobulin deficiency syndromes. In particular, SLE, rheumatoid arthritis, thyroiditis, and autoimmune hemolytic anemia have been reported in CVID as well as SIgAID [5, 12]. From our review, in pediatric SIgMID cases, autoimmune disease is distinctly uncommon (3.9%), compared to 12% in previously reported adult SIgMID cases (Table 4) [8].

Dysgammaglobulinemia has been reported with several GI conditions including steatorrhea, nodular lymphoid hypoplasia, Crohn’s disease, ulcerative colitis, amyloidosis, disaccharidase deficiencies, pernicious anemia, schlerosing cholangitis, celiac disease and protein losing enteropathies [4, 20, 50]. In particular, celiac disease has been reported in association with several primary immunodeficiencies including isolated severe SIgAID [52, 53] or reduced IgA levels (20—<60 mg/100 mL) [20, 54, 55], panhypogammaglobulinemia [53] and isolated combined IgA and IgM deficiency [53]. IgM deficiency has been more frequently reported—including 30 of 75 (37%) of adult cases, 5 of 5 childhood cases [20], 11 of 30 (37%) untreated adult patients [54], 8 of 11 untreated adult patients [55], 6 of 11 untreated, and 2 of 7 treated adult patients [55]. Studies based on catabolism and distribution of labeled IgM have not shown any difference in diet controlled untreated celiac disease [55]. Where reported, SIgMID did not correlate with any specific biochemical, hematologic or histologic abnormalities. Jejunal biopsies of affected patients were no different than those of celiac patients with normal immunoglobulin levels. There was no unusual risk of infection in these reported patients [55]. Of note, IgM levels returned to normal levels in most pediatric and adult patients following a gluten-restricted diet [20, 55]. In one study, mean pretreatment IgM level was 31.4 and the mean post treatment level 73.6, the difference being statistically significant (*P* = .0001) [20]. In those where the diet restriction was removed, IgM levels fell back to subnormal levels. It has been suggested that this secondary form of IgM deficiency is related to reduced synthesis from lymphoreticular dysfunction stimulated by gluten antigen exposure [54, 55].

The development of lymphoproliferative disorders and/or other malignancies is a concern with several primary immunodeficiencies, especially CVID [37]. In SIgMID the risk is relatively low in adults (2.6%) and negligible in children (0%). Pediatric cases of SIgMID also differ from adult cases in the absence of reported cases of angioedema, anaphylaxis, nasal polyps, bronchiectasis, and thyroid disease (Table 4). However, these conditions may become relevant concerns as children with SIgMID mature into adults. Vigilant followup and surveillance for these complications may therefore be warranted.

## 6. CONCLUSION

Pediatric SIgMID is a rare immunodeficiency with a prevalence of 0.03% in our symptomatic population. In our review of 51 pediatric patients with SIgMID, most patients presented with respiratory infections which, in general, were not severe or life threatening. The coexistence of autoimmune disease was rare, malignancies were not reported, and 4 fatalities were observed, 3 from fulminant infection, either meningitis or pneumonia. This report should alert clinicians to the possibility that SIgMID, although rare, may be the cause of recurrent respiratory infections in children. In addition, identification of patients with SIgMID may prevent some of the complications seen later in life with adult patients. Larger collaborative studies will better define the molecular genetics, pathogenesis, and clinical and immunologic phenotypes of this disorder in children.

## Figures and Tables

**Table 1:** Characteristics of previously reported pediatric cases of SIgMID. WNL: within normal limits, GN: glomerulonephritis, CMV: cytomegalovirus, AD: atopic dermatitis, N/A: not available, OM: otitis media, URI: upper respiratory infection.

Reference	Age of diagnosis (year)/sex	Presenting history	Serum IgM mg/dL	Serum IgG mg/dL	Serum IgA mg/dL	IgE IU/mL	Comment
[19]	9 male	Pyoderma, AD, diarrhea, cutaneous candidiasis, rudimentary auricles	1/4 of normal controls	WNL	WNL	N/A	⊕ Isohemagglutinin; Lack of IgM response to S. typhi; “O” antigen
[9]	4.5 mo female	Autoimmune anemia, GN, failure to thrive	13	2500	390	N/A	Died of PCP; diffuse lymphoid hypoplasia, thymus dysplasia
[6]	8 male	Meningococcal sepsis, Waterhouse- Friderichsen syndrome	4	560	70		Died from infection
[6]	5 male	Meningococcal meningitis	12	1120	190		Died from infection
[20]	3	Celiac disease	33	N/A	N/A	N/A	IgM level normalized after gluten free diet
[20]	8	Celiac disease	30	N/A	N/A	N/A	IgM level normalized after gluten free diet
[20]	10	Celiac disease	35	N/A	N/A	N/A	IgM level normalized after gluten free diet
[20]	13	Celiac disease	40	N/A	N/A	N/A	IgM level normalized after gluten free diet
[20]	2	Celiac disease	20	N/A	N/A	N/A	IgM level normalized after gluten free diet
[21]	5 male	Asymptomatic	35	820	224	N/A	Familial cases
[2]	3.75 male	N/A	7	495	172	N/A	Partial deletion of long arm of chromosome 18
[12]	6 male	Recurrent pneumonia, OM, AD, sinusitis	6	1250	950		Received IM gammaglobulin treatment, salmonella enteritis, pneumococcal sepsis
[16]	8.5 mo male	Pseudomonas pneumonia, recurrent OM	0	WNL	Mildly reduced		Died from infection
[18]	13 male	CMV hepatitis	26–29	N/A	N/A	N/A	
[14]	4	Meningitis	34	WNL	WNL	N/A	Low IgM hemagglutinin to meningococcus
[14]	1	Asymptomatic	36	WNL	WNL	N/A	Low IgM hemagglutinin to meningococcus
[22]	2.5 female	Recurrent OM, laryngitis, meningitis	8	530	48	N/A	Absent isohemagglutinin, chromosome #1 defect
[1]	3 patients, female <17	N/A	<23	WNL	WNL	N/A	
[1]	7 patients, male <16	N/A	<23	WNL	WNL	N/A	
[23]	12 female	Recurrent bronchitis, OM, pneumonia, bronchiectasis	34	686	87	N/A	No response to salmonella “H” antigen
[24]	Young male	N/A	8	1500	72	555
[10]	3 female	erythrodermic psoriasis	<4	680	125	340	OM, diarrhea, failure to thrive, recurrent respiratory infection, eosinophilia
[10]	9 male	AD	<4	900	153	8000	Failure to thrive, recurrent respiratory tract infection and pneumonia, skin infections, giardiasis, eosinophilia
[17]	16 female	Disseminated molluscum contagiosum	<4	2305	688	8900	AD, aphthous stomatitis, varicella, recurrent respiratory infections, eosinophilia, bronchiectasis
[25]	2.5 female	Failure to thrive, recurrent URI	37	869	45	N/A	
[25]	5.5 female	Failure to thrive, recurrent OM, eyelid and GI infections	38	1064	42	N/A	
[25]	8 male	Failure to thrive, recurrent OM, eyelid and GI infections	42	488	100	N/A	
[26]	1 mo	Pneumonia	<6	<350	N/A	N/A	Nonspecific IgG subclass deficiency
[26]	11 mo	Bronchitis, OM	<4	<350	N/A	N/A	
[26]	3	Bronchitis, OM	5	780	N/A	N/A	
[26]	2	OM	11	~2200	N/A	N/A	
[13]	1 mo	Meningitis, pneumonia, OM	6	717	27	<10	Allergic rhinitis
[13]	3	Bronchitis, OM	5	768	108	<10	Allergic rhinitis
[13]	2	OM	11	608	33	12	Allergic rhinitis
[13]	11 mo	OM, pneumonia	5	462	25	<10
[13]	11 mo	Bronchitis, OM	5	345	88	<10	Wheezing
[13]	8	OM, pneumonia	11	925	134	297	Allergic rhinitis, wheezing
[27]	10 male	Recurrent sinusitis, OM, pneumonia, chronic staphylococci blepharitis	23	900	86	N/A	
[28]	15 female	Chronic OM	<6	1170	356	N/A	22q11.2 chromosome deletion
[3]	11 male	Asthma	<5	NL	NL	N/A	Father with SIgMID
[29]	17 male	Russell-Silver Syndrome with recurrent URI	1	NL	NL	N/A	No response to pneumococcal vaccine
[30]	1.5 male	Recurrent OM	28	N/A	N/A	N/A	
[31]	7	Recurrent OM, pneumococcal pneumonia with emphysema	Deficient	NL	NL	Elevated	Lack of protective antibody response to polysaccharide and protein antigen, responded to IVIg
[31]	18 month	Asthma, recurrent OM, pseudomonal pneumonia, sepsis	Deficient	NL	NL	Elevated	
Mean	6 ± 4.7		16.5 ± 13.8	939 ± 580	183 ± 226	1814 ± 3509	

**Table 2:** Prevalence of presenting conditions in pediatric and adult patients with SIgMID.

Condition	Pediatric frequency percentage (*n* = 48)	Adult frequency [8] percentage (*n* = 191)
Pneumonia/lower respiratory tract infection	19.6	5.2
Otitis media	39.2	1.6
Bronchitis	9.8	3.1
Meningitis	7.8	0.5
Nonspecific bacterial respiratory infection	0	4.7
Upper respiratory infections	9.8	16.2
Failure to thrive	7.8	0
Other infections*	9.8	1.5
Allergic rhinitis	0	11.5
Vasomotor rhinitis	3.9	3.7
Nasal polyps	0	1.5
Asthma	7.8	16.2
Idiopathic angioedema	0	2.6
Idiopathic anaphylaxis	0	2.1
Gastrointestinal disease	13.7	4.2
Asymptomatic	3.9	3.1
Autoimmune disease	3.9	11.5
Skin disease	9.8	4.7
Bronchiectasis	0	2.6

*Nonrespiratory/nonmeningitis/nonskin.

**Table 3:** Immunologic abnormalities in SIgMID.

T cell functions	Increased IgM-specific T cell suppressor function [35, 40, 41]; normal T cell suppression function [42–44]
	Excessive isotype nonspecific T cell suppressive activity [40]; increased IgM specific suppressive T cell function [35]
	Defect in T cell help [10, 26]; normal T cell help [17, 42, 44, 45]
	Nonspecific T cell abnormalities [26]; normal T cell function [42]

B cell function	Defect in B cell differentiation into IgM-immunoglobulin secreting cells [40, 46, 47]

T and B cell enumeration and phenotype	Normal peripheral T and B cell phenotypes [42, 43, 47, 48]; increased CD8+ cells and inverted CD4/CD8 ratios [40]; increased CD4+ cells and decreased CD8+ cells [47]
	Reduced number of IgM secreting B cells [45, 46] with a failure of secreted mu mRNA synthesis [46]; normal surface IgM expression on B cells [10, 17, 35, 43, 47–49]; normal secreted mu mRNA synthesis [49]

Mitogen/antigen stimulation	Mitogen and antigen stimulated B cell proliferation assays with normal IgM responses [11, 42]; decreased antigen proliferation IgM responses [4, 17, 40, 41, 45–49]
	Deficient IgM responses to viral antigens and/or endotoxin containing vaccines and deficient isohemagglutinin antibodies [24]
	Failure to respond to antigen challenge with tetanus toxoid, pneumococcal vaccine, meningococcus vaccine, *Salmonella* O and H antigens, and typhus-paratyphus vaccine [10–12, 19, 42, 50]

Complement	No complement deficits [10]

Delayed hypersensitivity	Reduced delayed cutaneous hypersensitivity [10, 17]; normal delayed cutaneous hypersensitivity [11, 42]

Phagocytosis	Normal phagocytosis and killing of encapsulated bacteria [10]; nominally affected opsonification of yeast particles [11]; select opsonic defect against *Pseudomonas* [51]

**Table 4:** Frequencies of comorbid diseases in patients with SIgMID. (*n*/*a* = not applicable).

Disease state	Previously reported pediatric cases + 2 new cases percent (*n* = 51)	Previously reported adults cases [8] percent (*n* = 191)
Allergic rhinitis	9.8	12.6
Idiopathic angioedema	0	2.6
Idiopathic anaphylaxis	0	2.1
Asthma	18.7	24
Autoimmune disease*	3.9	12
Bronchiectasis	0	2.6
Failure to Thrive	9.8	N/A
Gastrointestinal disease**	15.7	12
Malignancy	0	2.6
Meningitis	7.8	0.5
Nasal polyps	0	0.5
Other infections***	9.8	1.6
Recurrent respiratory infections	74.5	32.5
Skin disease	11.8	4.7
Thyroid disease	0	0.5
Vasomotor rhinitis	3.9	37
Fatalities	7.8	0
Asymptomatic	3.9	3.1

*Autoimmune thyroiditis, autoimmune anemia, autoimmune glomerulonephritis, rheumatoid arthritis, SLE. **Includes celiac disease, inflammatory bowel disease, diarrhea syndromes. ***Nonrespiratory/nonmeningitis/nonskin.
